# *Gardenia*–*Astragalus*–*Crataegus* Extract Alleviates Carbon Tetrachloride-Induced Liver Injury in Mice Through Antioxidant and Apoptosis Pathways

**DOI:** 10.3390/antiox15060701

**Published:** 2026-06-01

**Authors:** Guangpei Xu, Yanfei He, Xin He, Ping Jiang, Chuanbo Sun, Xinghua Zhao

**Affiliations:** 1 College of Veterinary Medicine, Hebei Agricultural University, Baoding 071000, China; guangpeixu@163.com (G.X.); dyhexin@hebau.edu.cn (X.H.); 2 College of Life and Health, West Anhui University, Lu’an 237000, China; 341281a00ek.cdb@sina.cn (Y.H.); jiangping0550@126.com (P.J.)

**Keywords:** *Gardenia–Astragalus–Crataegus* extract, liver injury, oxidative stress, PI3K-Akt, MAPK

## Abstract

The incidence of liver injury has been increasing year by year, potentially progressing to liver fibrosis, cirrhosis, and even hepatocellular carcinoma, posing a threat to human health. *Gardenia*, *Astragalus*, and Hawthorn are commonly used natural products with dual purposes as both medicine and food. While extensive research has been conducted on their individual pharmacological activities, systematic studies on the synergistic effects and efficacy evaluation of their combined use in liver protection are still lacking. This study aimed to investigate the protective effects and underlying mechanisms of the compound *Gardenia* and Scutellaria extract (GACE) on CCl4-induced liver injury in mice. The composition and content of GACE were analyzed by HPLC. Serum biochemical, inflammatory, and hepatic oxidative stress indicators were detected using assay kits. Liver pathological changes were examined by HE staining, while apoptosis was observed via TUNEL staining. Transcriptomic and metabolomic sequencing were employed to analyze the potential mechanisms of CCl4 in alleviating liver injury. Finally, Western blotting was performed to validate the analytical results. The results demonstrated that CCl4 significantly upregulated the levels of pro-inflammatory cytokines IL-6, IL-12 and IL-2 (*p* < 0.05), downregulated the level of anti-inflammatory cytokine IL-10 (*p* < 0.05), and caused markedly elevated serum ALT and AST levels (*p* < 0.01). The hepatic lobule structure was nearly obliterated in liver tissue, with disordered arrangement of hepatic cords and numerous vacuoles appearing in hepatocytes. The TUNEL staining positive cell rate increased significantly compared with the blank group (*p* < 0.001). Meanwhile, CCl4 also significantly inhibited SOD and GSH-Px activities in liver tissue (*p* < 0.05), while markedly increasing MDA levels (*p* < 0.05). GACE significantly alleviated liver tissue damage and reduced hepatocyte apoptosis (*p* < 0.05). It markedly enhanced SOD and GSH-Px activities in liver tissue while decreasing ALT, AST, MDA, IL-6, IL-12, and IL-2 levels (*p* < 0.05), thereby mitigating hepatic oxidative damage and inflammatory responses. Integrated transcriptomic and metabolomic analyses revealed that the PI3K-Akt and MAPK signaling pathways play crucial roles in mediating GACE’s therapeutic effects against liver injury. Further validation demonstrated that GACE can attenuate CCl4-induced liver injury by inhibiting the PI3K-Akt and MAPK pathways.

## 1. Introduction

Liver injury refers to abnormal liver function caused by various pathogenic factors, such as chemical toxins, immune-mediated injury, drug-induced damage, and alcohol abuse [1]. In recent years, the global incidence of liver injury has been steadily rising, often accompanied by severe complications including liver failure, hepatic fibrosis, and even hepatocellular carcinoma, making it a significant public health issue affecting population health [2]. In the preclinical evaluation of hepatoprotective drugs, chemically induced models are widely employed, among which carbon tetrachloride (CCl4) is one of the most commonly used hepatotoxic agents to simulate liver injury in experimental animals [3]. CCL4 is primarily activated by hepatic cytochrome P450 oxidase to generate trichloromethyl radicals, which covalently bind to unsaturated lipids in cell membranes, triggering lipid peroxidation, damaging intracellular proteins, lipids, and DNA, and ultimately leading to mitochondrial dysfunction and hepatocyte apoptosis [4]. In addition, CCL4 attacks phospholipids in the hepatocyte membrane, causing lipid peroxidation and disrupting its membrane structure, thereby damaging hepatocytes. It also releases hepatic type IV collagenase to degrade type IV collagen in the liver, promoting the formation of hepatic fibrosis [5].

Traditional Chinese medicine, as a traditional natural medicine, has accumulated rich practical experience in the prevention and treatment of liver diseases. It possesses unique advantages such as multi-target, multi-pathway, and low side effects, and has gradually become a hot research direction in the development of hepatoprotective drugs in recent years [6,7]. Research reports that 246 patients with non-alcoholic fatty liver disease (NAFLD) and abnormal liver function were treated with Qinggan Huayu Jiedu Capsules, and it was found that the treatment significantly reduced the liver fat content and liver enzyme levels, improved the gut microbiota, and exhibited good clinical potential in treating non-alcoholic fatty liver disease (NAFLD) [8].

*Gardenia jasminoides* is the mature fruit of the *Gardenia* plant in the Rubiaceae family. It is a traditional Chinese medicinal and edible substance, commonly used for clearing heat, removing dampness, and detoxifying. Modern pharmacological studies have shown that *Gardenia jasminoides* possesses various pharmacological effects, including anti-inflammatory, antioxidant, anti-fatigue, and antithrombotic activities [9]. Studies have found that *Gardenia jasminoides* can enhance anti-oxidative stress and anti-inflammatory capabilities by regulating the Nrf2/AMPK/mTOR signaling pathway, thereby attenuating damage from non-alcoholic fatty liver disease [10]. In Tangpradubkiat’s study, *Gardenia* exerted anti-inflammatory and antioxidant effects by reducing serum liver enzymes and inflammatory factors, as well as increasing antioxidant enzyme activity, thus alleviating acetaminophen-induced liver injury in mice [11]. Geniposide, the main active component of *Gardenia*, possesses functions such as alleviating inflammation, antioxidant activity, immune regulation, improving lipid metabolism, and preventing thrombosis [12,13]. Studies have shown that geniposide exerts therapeutic effects on intrahepatic cholestasis by modulating bile secretion pathways and glutathione pathways, as well as regulating the expression of *ABCG5*, *NCEH1*, *OAT3*, and *GST* genes [14]. Furthermore, the active component geniposidic acid in *Gardenia jasminoides* can significantly ameliorate hepatic lipid accumulation, oxidative stress, inflammatory infiltration, and fibrosis in mice fed a high-fat diet or a methionine–choline-deficient diet.

*Astragalus radix*, the dried root of plants in the genus *Astragalus* (Fabaceae), possesses properties that tonify Qi, promote diuresis, and enhance immune function. Modern pharmacological studies have confirmed its various therapeutic characteristics, exhibiting a wide range of pharmacological effects, including diuretic, anti-aging, antihypertensive, immune-enhancing, hepatoprotective, and anti-stress activities [15,16]. Research indicates that *Astragalus* and its active constituents can promote liver regeneration by upregulating AQP9 expression in hepatocytes to increase gluconeogenesis and reduce oxidative stress [17]. Astragaloside IV, the primary active component of *Astragalus*, has potential in the prevention and treatment of various liver diseases, including multifactorial liver injury, metabolic associated fatty liver disease, liver fibrosis, and hepatocellular carcinoma [18]. Astragaloside IV can effectively alleviate fibrosis-induced dysfunction of major tissues or organs such as the heart, lung, kidney, and liver by regulating the signal transduction of reactive oxygen species/caspase-1/gasdermin D, transforming growth factor-β/Smads, Wnt/β-catenin, and sirtuin 1-nuclear factor-κB [19]. In addition, *Astragalus* polysaccharidesye, as a main active component of *Astragalus* membranaceus, can exert significant anti-hepatocellular carcinoma effects through various biological actions, including promoting apoptosis, inhibiting proliferation, suppressing epithelial–mesenchymal transition, regulating autophagy, and modulating immune responses [20]. *Crataegus pinnatifida*, as the fruit of the Rosaceae family plant, has functions of promoting digestion, eliminating blood stasis, and regulating lipid metabolism. Modern pharmacological studies have confirmed its anti-inflammatory, hypoglycemic, antihypertensive, and lipid-lowering effects [21]. Research has shown that hawthorn can reduce free fatty acid-induced lipid accumulation in HepG2 cells by regulating the AMPK and NF-κB signaling pathways, indicating its potential value in the treatment of fatty liver disease [22]. Additionally, hawthorn polysaccharides alter the gut environment by modulating the gut microbiota, stimulating the production of short-chain fatty acids, and alleviating colitis, thereby contributing to the improvement of nonalcoholic fatty liver [23].

The *Jin Gui Yao Lue* records: “In treating disease before it arises, when one sees a disorder of the liver, one knows that the liver can transmit to the spleen, and should therefore first strengthen the spleen.” This means that while treating liver disease, one should also pay attention to regulating and supplementing the spleen, so that the spleen’s healthy Qi is fortified, preventing the spread of liver disease. This is the method of soothing the liver and strengthening the spleen commonly adopted in traditional Chinese medicine for treating liver diseases. *Gardenia* excels at clearing heat from the heart, liver, and Triple Energizer, while also promoting the resolution of damp-heat from the liver and gallbladder; *Astragalus* is an important Qi-supplementing herb, boosting Qi and strengthening the spleen, protecting the liver and benefiting the gallbladder; *Crataegus* harmonizes the spleen and stomach, dissipates stasis and moves stagnation. The combined use of these three herbs achieves the effects of supplementing Qi and strengthening the spleen, clearing the liver and discharging heat, and harmonizing both Qi and blood, which aligns with the core pathogenesis of liver injury. Based on this theory, we formulated a *Gardenia*, *Astragalus*, and *Crataegus* extract (GACE) to evaluate its therapeutic effects in a mouse model of acute liver injury induced by carbon tetrachloride (CCl4), and to elucidate its mechanism of action, thereby providing experimental evidence and theoretical support for the development of novel hepatoprotective drugs.

## 2. Materials and Methods

### 2.1. Reagents and Materials

The following instruments, reagents, and materials were used in this study: Waters-2695 HPLC System (Waters, Milford, MA, USA); Thermo Scientific UltiMate 3000 UHPLC System (Thermo Fisher Scientific, Waltham, MA, USA); JA2003 Analytical Balance (Shanghai Sunny Hengping Scientific Instrument Co., Shanghai, China); Five-part Hematology Analyzer (Shenzhen Mindray Bio-Medical Electronics Co., Shenzhen, China); MB-580 Microplate Reader (Shenzhen Huisong Technology Development Co., Shenzhen, China); ALT and AST Detection Kits (Nanjing Jiancheng Bioengineering Institute, Nanjing, China); ELISA kits for IL-2, IL-6, IL-10, IL-12, TNF-α, and CCL8 (Wuhan Boster Biological Technology Ltd., Wuhan, China); and *Gardenia* jasminoides (Batch No.: 100017903711, produced in Fujian, China), Astragali Radix (Batch No.: 100076340644, produced in Gansu, China), and *Crataegus* pinnatifida (Batch No.: 100017405838, produced in Shandong, China) sourced from Nanjing Tongrentang Pharmaceutical Co., Ltd., Nanjing, China. All medicinal materials were identified by Professor Shi Wanyu (Hebei Agricultural University, Baoding, China) in accordance with the Chinese Pharmacopoeia (2020 edition), and stored at the Traditional Chinese Medicine Laboratory of West Anhui University (wxxy20240510).

### 2.2. Preparation and Fingerprint Analysis of GACE

Precisely weigh 1000 g each of *Gardenia jasminoides* J., *Astragalus membranaceus*, and *Crataegus pinnatifida*. At laboratory scale, perform reflux extraction twice with 10-fold volume of 75% ethanol for 2 h each time. Filter the extracts, concentrate under reduced pressure using a rotary evaporator to obtain dry powder, and mix to yield 980.2 g of the compound *Gardenia*–*Astragalus* extract (GACE), with a yield rate of 32.67%. The concentrations of geniposide and chlorogenic acid in GACE were quantified using a Waters-2695 HPLC system equipped with a diode array detector (DAD). Chromatographic separation was achieved on a Diamond C18 column (5 μm, 250 mm × 4.6 mm) maintained at 35 °C. The mobile phase consisted of acetonitrile (A) and 0.1% formic acid (B), with a gradient elution program as follows: 0–5 min, 2.5% A; 5–50 min, linear increase from 2.5% to 48% A; 50–55 min, linear increase to 100% A; 55–57 min, 100% A; and 57–58 min, decrease back to 2.5% A. The injection volume was 10 μL, and detection was carried out using a photodiode array (PDA) detector (Waters, Milford, MA, USA).

### 2.3. Animal Treatment

Female ICR mice (6 weeks old, weighing 18–22 g) were obtained from Henan Skbess Biotechnology Co., Ltd. (Henan, China) (Production License No.: SCXK (Yu) 2020–0005) and acclimated for 7 days under specific pathogen-free (SPF) conditions at 22 °C with 55–65% relative humidity, with ad libitum access to food and water. All animal procedures adhered to the Chinese guidelines for the care and use of laboratory animals and were approved by the Animal Care and Use Committee of West Anhui University (Approval No. 202504005, approved on 7 April 2025). The mice were randomly assigned to six groups (*n* = 6 per group): Control, Model, Positive Control (Silibinin, SIL), and low- (1.5 g/kg), medium- (3 g/kg), and high-dose (6 g/kg) GACE treatment groups. From days 1 to 10, GACE-treated groups received their respective doses via intragastric gavage at 0.2 mL/10 g body weight, while the Positive Control group received SIL at 100 mg/kg, and the Control and Model groups were administered saline. From days 7 to 9, all groups except the Control group were intraperitoneally injected with 0.3% CCl4 dissolved in olive oil (10 mL/kg) [24,25], administered one hour after the final gavage; the Control group received olive oil alone. After the experiment, mice were anesthetized with 1% sodium pentobarbital (60 mg/kg, intraperitoneally), and serum, liver tissues, and cecal contents were collected for subsequent analysis.

### 2.4. Biochemical Assays

Blood samples were collected via orbital sinus puncture into anticoagulant tubes, and complete blood counts were performed using hematology analyzer (Mindray, Shenzhen, China). Serum levels of alanine aminotransferase (ALT) and aspartate aminotransferase (AST) were determined using commercial kits from Nanjing Jiancheng Bioengineering Institute, following the Reitman-Frankel method. Serum cytokines, including IL-10, IL-6, TNF-α, IL-2, IL-12, and CCL8, were quantified using ELISA kits obtained from Wuhan Boster Biological Technology Ltd. For hepatic biochemical analysis, liver tissues were homogenized, and the homogenates were centrifuged to collect supernatants, which were subsequently analyzed for superoxide dismutase (SOD), malondialdehyde (MDA), and glutathione peroxidase (GSH-Px) levels using corresponding assay kits from Nanjing Jiancheng.

### 2.5. Histological Analysis

Liver tissues were fixed in 4% paraformaldehyde, embedded in paraffin, sectioned at a thickness of 5 μm, and stained with hematoxylin and eosin (H&E) for histopathological examination under a light microscope. To assess the degree of necrosis after acute liver injury, We refer to the Knodell scoring system for intralobular hepatocyte degeneration and focal necrosis (scores 0–4). Each sample was independently scored by three pathologists who were blinded to the treatment and untreated control groups. The scoring system was as follows [26]: Grade 0, no pathological changes; Grade 1, hepatocyte degeneration with only rare necrotic foci; Grade 2, mild centrilobular necrosis in small areas around the central veins; Grade 3, mild centrilobular necrotic areas, more severe than Grade 2; and Grade 4, centrilobular necrosis more severe than Grade 3. For TUNEL staining, tissue sections were deparaffinized, treated with proteinase K for 20 min, rinsed with PBS, counterstained with DAPI, and examined using fluorescence microscopy to detect apoptotic cells exhibiting green fluorescence. Quantification of apoptotic cells was performed using ImageJ software (version 6.0).

### 2.6. Transcriptomic and Weighted Gene Co-Expression Network Analysis (WGCNA)

Total RNA was extracted from liver tissues, quantified, and assessed for quality prior to library preparation and sequencing on the Illumina HiSeq X Ten platform (San Diego, CA, USA). Raw sequencing reads were processed using Trimmomatic v0.32 to remove low-quality reads and adapters, yielding clean reads that were aligned to the reference transcriptome using HISAT2 v2.2.1 and Bowtie2 v2.3.2. Gene expression levels were quantified as fragments per kilobase of transcript per million mapped reads (FPKM) for subsequent differential expression analysis. WGCNA was conducted using the R package “WGCNA” (1.5) to construct co-expression networks, identify gene modules significantly associated with GACE treatment, and filter out genes with low expression (FPKM < 0.5). Outlier samples were identified through hierarchical clustering. Genes within the selected modules were intersected with differentially expressed genes (DEGs), and the overlapping gene set was subjected to KEGG pathway enrichment analysis.

### 2.7. Metabolomics Analysis (UPLC-ESI-QTOFMS Analysis and Data Processing)

Data were acquired using a Thermo Scientific UltiMate 3000 UHPLC system (Waltham, MA, USA) coupled with a mass spectrometer. Chromatographic separation was carried out on an ACQUITY UPLC BEH C18 column (100 mm × 2.1 mm, 1.8 μm; Waters, Wilmslow, UK) maintained at 35 °C, with a flow rate of 0.4 mL/min. The mobile phase consisted of phase A (water containing 0.1% formic acid) and phase B (acetonitrile containing 0.1% formic acid). Raw mass spectrometry data were converted to mzXML format using MSConvert tool (ProteoWizard software suite; version 3.0.22048). Peak extraction and quality control were performed using XCMS, and adduct ion annotation was conducted with CAMERA. Primary metabolite identification was accomplished using metaX software (1.4.2) by integrating MS1 data and MS2 fragmentation patterns, which were matched against an in-house standard database. Candidate metabolites were further annotated using public databases such as HMDB and KEGG to interpret their physicochemical characteristics and biological functions. Differential metabolites were then quantified and statistically screened using metaX.

### 2.8. Western Blot Validation of Related Protein Expression

Liver tissues were minced and lysed in RIPA buffer containing PMSF (a protease inhibitor) on ice for 30 min, followed by centrifugation to collect the supernatants. Total protein concentration was determined using the BCA assay. Proteins were separated by 12% SDS-PAGE, initially at 70 V for 30 min in the stacking gel and then at 110 V for 60 min in the resolving gel, before being transferred to PVDF membranes at 300 mA for 90 min. The membranes were blocked with blocking buffer for 2 h at room temperature, washed with TBST, and incubated overnight at 4 °C with primary antibodies diluted at 1:10,000. After removal of the primary antibodies and thorough washing, the membranes were incubated with secondary antibodies (1:10,000 dilution) for 2 h at room temperature. Protein bands were visualized following additional washes, and densitometric analysis was conducted using ImageJ software. Relative protein expression levels were calculated as the ratio of the target protein band intensity to that of the internal reference protein.

### 2.9. Statistical Analysis

Data are expressed as mean ± standard deviation (Mean ± SD). Statistical analyses were conducted using one-way analysis of variance (ANOVA) in SPSS version 21.0, with a *p*-value < 0.05 considered statistically significant.

## 3. Results

### 3.1. Establishment of Fingerprint and Determination of GACE

**Figure 1:**
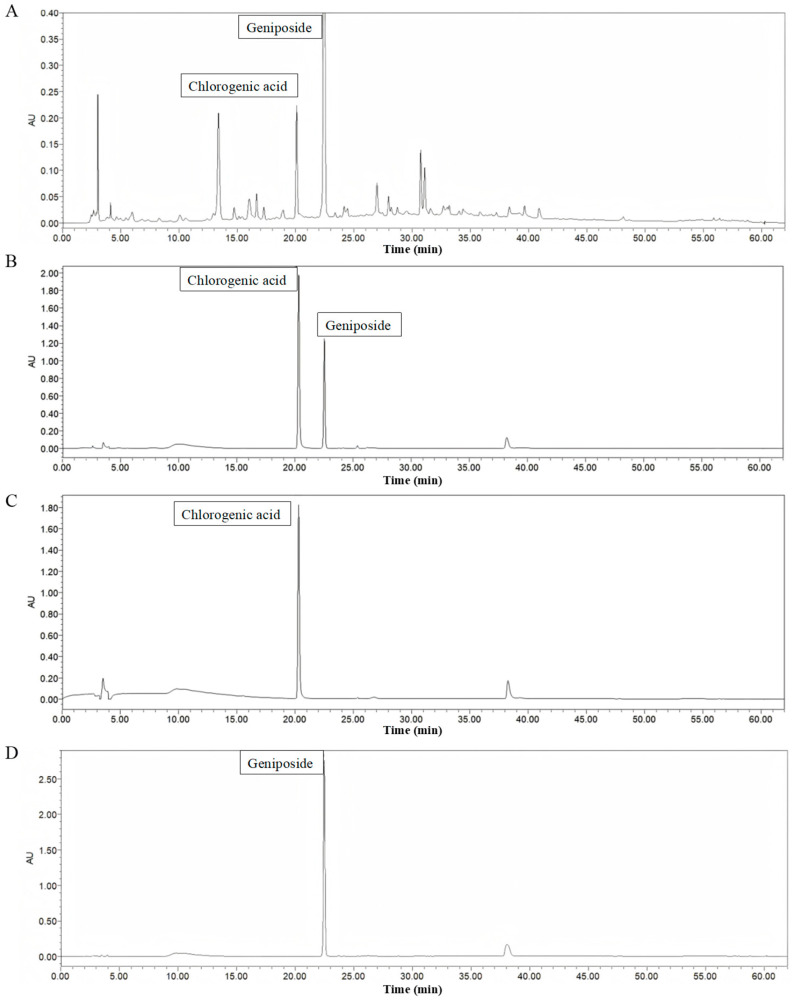
HPLC chromatogram of GACE. (**A**) Sample chromatogram, (**B**) chromatogram of mixed standard solution of chlorogenic acid and geniposide, (**C**) chlorogenic acid chromatogram, (**D**) geniposide chromatogram.

The HPLC fingerprint of GACE was established (Figure 1A), along with the chromatogram of the mixed standard solution of chlorogenic acid and geniposide (Figure 1B), and the chromatograms of chlorogenic acid and geniposide (Figure 1C,D). Based on this chromatographic method, the standard curve A was constructed with the concentration of chlorogenic acid as the horizontal axis and the peak area of chlorogenic acid as the vertical axis: y = 2 × 10^7^x − 2 × 10^6^ (R^2^ = 0.9986), where x is the concentration of chlorogenic acid. The standard curve B was constructed with the concentration of geniposide as the horizontal axis and the peak area of geniposide as the vertical axis: y = 1 × 10^7^x + 1 × 10^6^ (R^2^ = 0.9971), where x is the concentration of geniposide. The concentration of chlorogenic acid in GACE was calculated as 0.596 mg/g based on Standard Curve A, and the concentration of geniposide was calculated as 0.872 mg/g based on Standard Curve B.

### 3.2. Effects of GACE on Inflammatory Cells and Cytokines in Mice with Liver Injury

**Figure 2:**
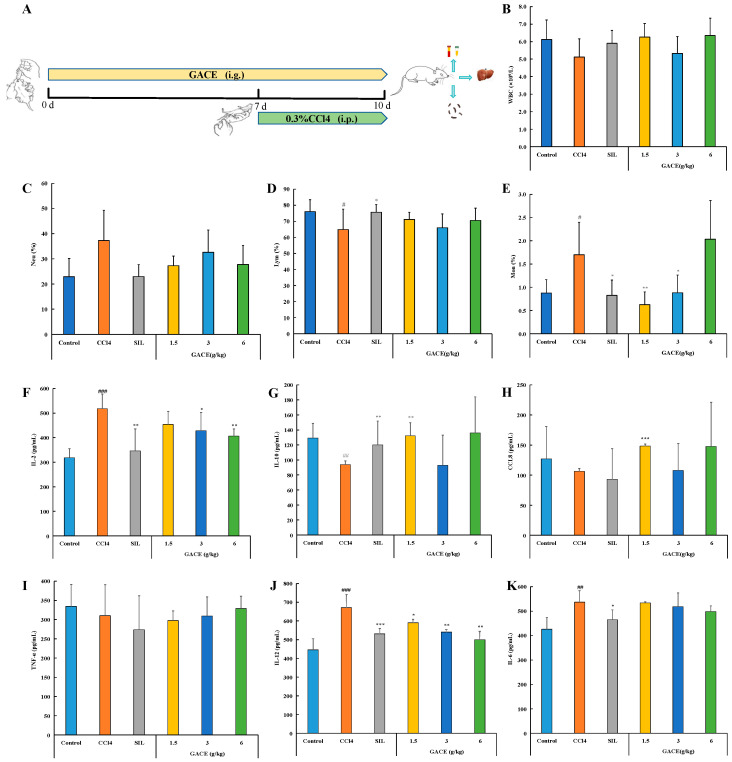
Effects of GACE on Inflammatory Cells and Cytokines in Mice with CCl4-Induced Liver Injury. (**A**) Experimental timeline, (**B**) blood leukocytes in mice, (**C**) blood neutrophils in mice, (**D**) blood lymphocytes in mice, (**E**) blood monocytes in mice, (**F**) serum IL-2 levels in mice, (**G**) serum IL-10 levels in mice, (**H**) serum CCL8 levels in mice, (**I**) serum TNF-α levels in mice, (**J**) serum IL-12 levels in mice, (**K**) serum IL-6 levels in mice. (*n* = 6 in each group; compared with the control group, # *p* < 0.05, ## *p* < 0.01, ### *p* < 0.001 in the model group; compared with the model group, * *p* < 0.05, ** *p* < 0.01, *** *p* < 0.001).

The flow chart of the mouse experiment and the drug administration methods are shown in Figure 2A. Compared with the control group, the model group exhibited significantly elevated percentages of Neu (Neutrophils) and Mon (Monocytes) in the blood (Figure 2C,E). In contrast, the low- and medium-dose GACE groups showed marked reductions in Neu and Mon percentages compared to the model group (*p* < 0.05) (Figure 2C,E). The percentage of Lym (Lymphocytes) was significantly lower in the model group than in the control group (*p* < 0.05), whereas the low-dose GACE group demonstrated a significant increase in Lym percentage compared to the model group (*p* < 0.05) (Figure 2D). ELISA results further revealed that CCl4 significantly upregulated the expression of pro-inflammatory cytokines, including IL-6, IL-12, and IL-2 (*p* < 0.05) (Figure 2F,J,K), while simultaneously downregulating the anti-inflammatory cytokine IL-10 (*p* < 0.05) (Figure 2G). GACE significantly suppressed CCl_4_-induced elevation of pro-inflammatory cytokines and restored the anti-inflammatory cytokine. These changes correlate with reduced circulating neutrophils and monocytes, indicating that GACE modulates systemic and local inflammation.

### 3.3. GACE Attenuates CCl4-Induced Liver Injury

**Figure 3:**
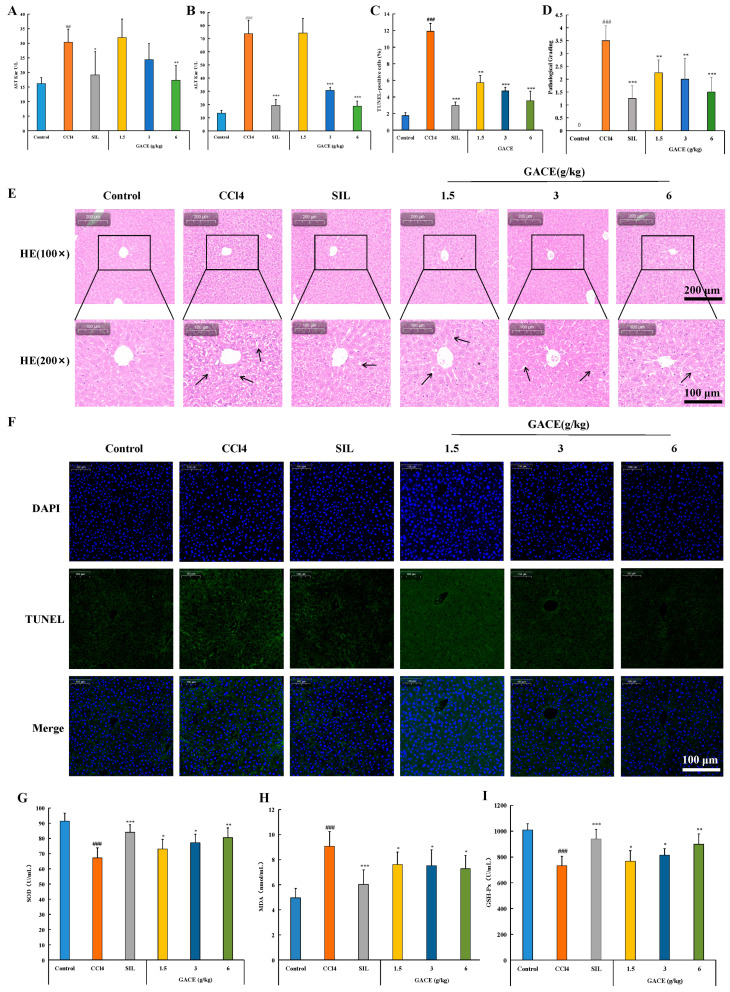
GACE alleviated CCl_4_-induced liver injury in mice. (**A**) Serum AST levels, (**B**) serum ALT levels, (**C**) rate of TUNEL-positive cells in liver tissue, (**D**) pathological grading, (**E**) HE staining of liver tissue (the arrow indicates the clearly damaged area of liver cells, the rectangular box in the low-magnification image delineates the area that is enlarged and presented in the high‑magnification image), (**F**) TUNEL staining of liver tissue, (**G**) hepatic SOD levels, (**H**) hepatic MDA levels, (**I**) hepatic GSH-Px activity. (*n* = 6 in each group; compared with the control group, ## *p* < 0.01, ### *p* < 0.001 in the model group; compared with the model group, * *p* < 0.05, ** *p* < 0.01, *** *p* < 0.001).

Serum ALT and AST levels were significantly elevated in the model group compared to the control group (*p* < 0.05), indicating severe hepatocellular damage (Figure 3A,B), whereas GACE administration effectively reduced these enzyme levels, with the high-dose group exhibiting the most pronounced decreases (*p* < 0.01, *p* < 0.001). H&E staining revealed that CCl4 caused extensive liver injury, including disrupted lobular architecture, disorganized hepatic cords, and numerous intracellular vacuoles (arrowed), while both GACE and SIL treatments significantly alleviated these histopathological changes (Figure 3D). The pathological scoring criteria showed that, compared with the control group, CCl4 caused significant liver injury (*p* < 0.001), and after GACE treatment, the liver tissue damage was significantly improved. Oxidative stress assays further demonstrated that CCl4 induced severe hepatic oxidative damage, as evidenced by significantly reduced SOD and GSH-Px activities and elevated MDA levels in the model group compared to the control group (*p* < 0.05). GACE treatment dose-dependently restored SOD and GSH-Px activities and decreased MDA levels, all with statistical significance (Figure 3G–I). TUNEL staining showed a markedly higher number of apoptotic hepatocytes in the model group versus the control group (*p* < 0.001), whereas treatment with SIL and all doses of GACE significantly reduced apoptosis, as reflected by decreased TUNEL-positive rates (Figure 3C,F). These findings collectively confirm that GACE effectively ameliorates CCl4-induced hepatic injury, oxidative stress, and hepatocyte apoptosis.

### 3.4. Transcriptome Combined with WGCNA for Elucidating Hepatoprotective Mechanism of GACE

**Figure 4:**
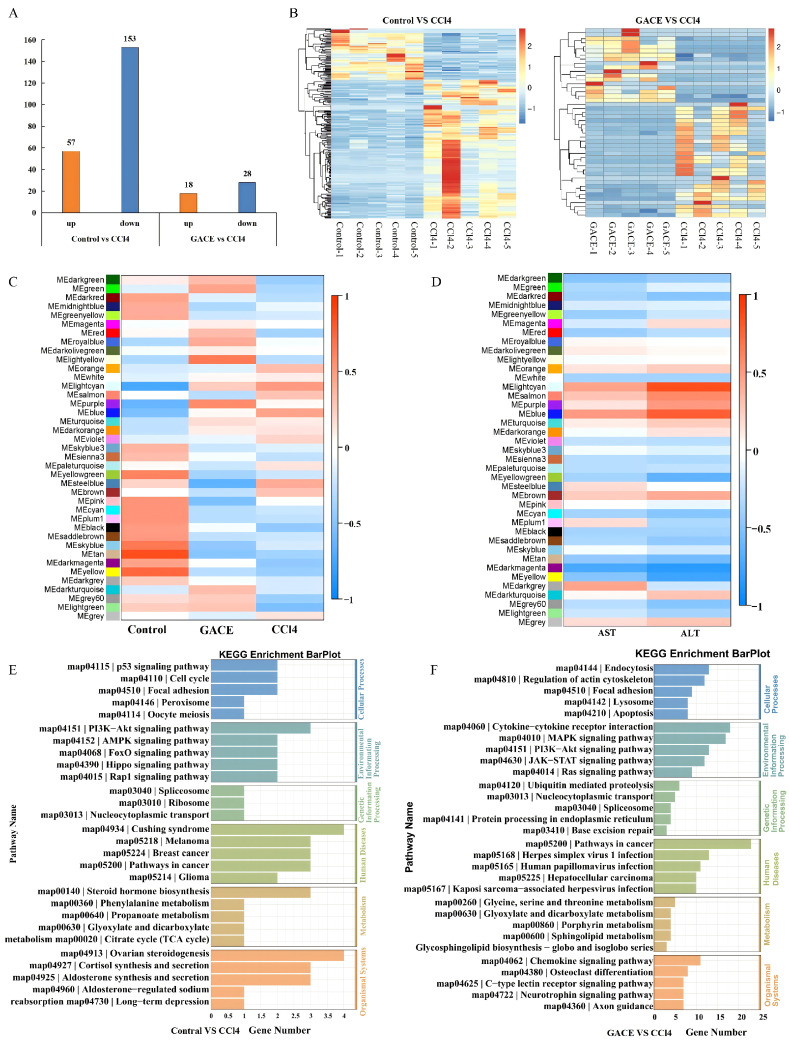
Transcriptomic Analysis of Liver Tissue in CCl4-induced Liver Injury Mice Treated with GACE. (**A**) Volcano plot of differentially expressed genes, (**B**) heatmap of differentially expressed gene enrichment, (**C**) heatmap of module-condition correlation analysis, (**D**) heatmap of module-trait relationship analysis, (**E**) KEGG enrichment pathway diagram of the model group, (**F**) KEGG enrichment pathway diagram of the GACE group. (*n* = 5 in each group).

Transcriptome sequencing analysis, using thresholds of fold change (FC) ≥ 2 or ≤0.5 (|log2FC| ≥ 1) and q < 0.05, identified 57 upregulated and 153 downregulated genes in the model group compared with the control group, while the GACE group, in comparison to the model group, exhibited 18 upregulated and 28 downregulated genes. Cluster analysis of these DEGs revealed that the number of highly expressed hepatic genes was significantly lower in the control and GACE groups compared to the model group (Figure 4A,B), indicating that GACE modulates gene expression altered by CCl4-induced liver injury. WGCNA of trait–module correlations revealed that the MEtan, MEyellow, and MEyellowgreen modules were positively correlated with the model group, while MEsteelblue and MEpink were negatively correlated and MElightyellow and MEpurple were positively correlated with the GACE group (Figure 4C). Furthermore, the Medarkmagenta module was negatively correlated with AST levels, whereas ALT was negatively correlated with the Medarkmagenta, MEyellow, and MEyellowgreen modules and positively correlated with the Melightcyan and Meblue modules (Figure 4D). These findings suggest that genes within these specific modules may serve as potential targets associated with CCl4-induced liver injury and its mitigation by GACE treatment. Functional enrichment analysis of genes within the relevant modules demonstrated that those associated with both the model group and the GACE group were significantly enriched in the PI3K-AKT signaling pathway (Figure 4E,F), suggesting that GACE may exert its hepatoprotective effects, at least in part, by modulating the PI3K-AKT signaling pathway in mice.

### 3.5. Metabolomic Analysis of GACE in CCl4-Induced Liver Injury in Mice

**Figure 5:**
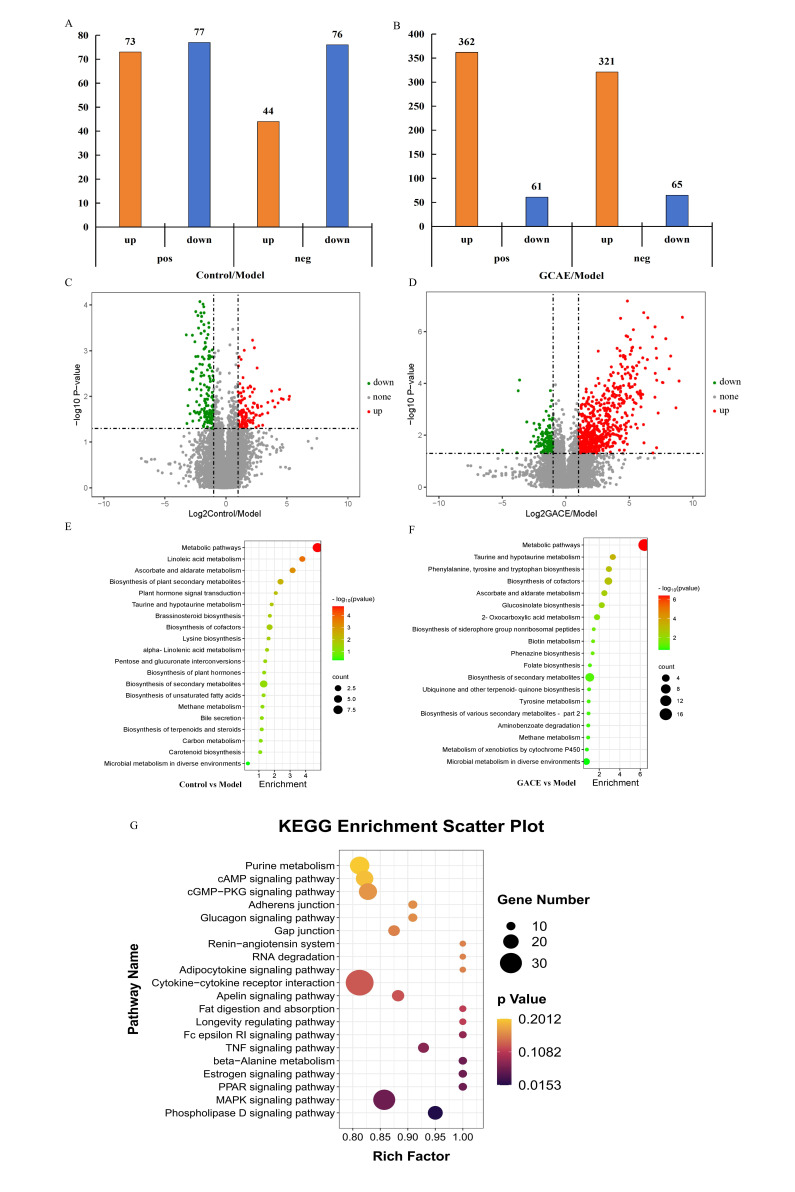
Metabolomic analysis of GACE on CCl4-induced liver injury in mice. (**A**) Column chart of differential metabolites between control and model group in positive and negative ion modes, (**B**) Column chart of differential metabolites between GACE and model group in positive and negative ion modes, (**C**) Volcanic map of differential metabolites between control and model group, (**D**) Volcanic map of differential metabolites between GACE and model group, (**E**) KEGG metabolic pathway bubble plots for control and model group, (**F**) KEGG metabolic pathway bubble plots for GACE and model group, (**G**) Bubble chart of the top 20 metabolic pathways enriched by KEGG. (*n* = 5 in each group).

As shown in Figure 5A,B, compared with the control group, the model group exhibited 73 upregulated and 77 downregulated metabolites in positive ion mode, and 44 upregulated and 76 downregulated metabolites in negative ion mode; in contrast, compared with the model group, the GACE group showed 362 upregulated and 61 downregulated metabolites in positive ion mode, and 321 upregulated and 65 downregulated metabolites in negative ion mode (Figure 5C,D).

Screening of secondary differential metabolites followed by KEGG enrichment analysis revealed that the differential metabolites among the control, model, and GACE groups were mainly enriched in ascorbate and aldarate metabolism, as well as cofactor biosynthesis pathways (Figure 5E,F), while further signaling pathway enrichment analysis indicated that these metabolites were primarily involved in cytokine–cytokine receptor interaction and the MAPK signaling pathway (Figure 5G), collectively suggesting that GACE can significantly regulate metabolic processes in mice.

### 3.6. GACE Protects Against CCl4-Induced Liver Injury in Mice by Regulating PI3K/AKT and MAPK Pathways

**Figure 6:**
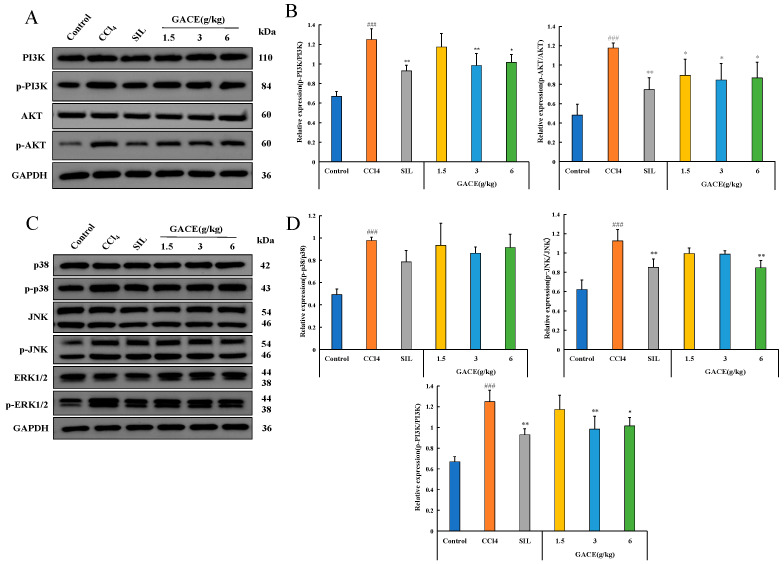
Effect of GACE on the PI3K/MAPK pathway in CCl4-Induced liver Injury in mice. (**A**) Expression levels of PI3K and AKT proteins in mouse liver tissue, (**B**) mean optical density values of PI3K/AKT in liver tissue (*n* = 3; the values are presented as mean ± SD), (**C**) expression levels of p-38, JNK, and ERK pathway-related proteins in mouse liver tissue, (**D**) mean optical density values of MAPK pathway-related proteins in liver tissue (*n* = 3 in each group; the values are presented as mean ± SD; compared with the control group, ^###^ *p* < 0.001 in the model group; compared with the model group, * *p* < 0.05, ** *p* < 0.01).

Figure 6 presents there was no significant change in the total protein levels of PI3K, AKT, p38, JNK, and ERK1/2 in each group (*p * > 0.05). GACE administration markedly reduced the phosphorylation levels of these proteins, showing a similar inhibitory effect as observed in the SIL group (Figure 6B,D). These findings suggest that GACE mitigates CCl4-induced liver injury in vivo by suppressing the activation of the PI3K/AKT and MAPK signaling pathways.

## 4. Discussion

The liver, a crucial organ responsible for metabolism and detoxification, can suffer dysfunction due to various factors such as chemical exposure, alcohol consumption, and viral infections; if not promptly treated, liver injury may progress to more severe conditions like hepatic fibrosis, cirrhosis, or hepatocellular carcinoma (HCC). According to modern medical insights, liver injury is closely associated with inflammatory responses, oxidative stress, and endoplasmic reticulum stress [27]. Research has shown that CCl4 is metabolically activated by hepatic cytochrome P450 enzymes to generate trichloromethyl and chlorine radicals, which initiate lipid peroxidation of hepatocyte membrane phospholipids. The resulting excessive reactive oxygen species (ROS) covalently bind to membrane lipids and proteins, thereby disrupting cellular membrane structure and function [28]. This process forms the basis of a widely accepted and reliable model for inducing liver injury in studies evaluating hepatoprotective agents. In our study, the CCl4-induced liver injury model showed markedly elevated serum and hepatic ALT and AST levels in the model group compared to the control group, with histopathological examination and TUNEL staining confirming significant hepatic damage.

Oxidative stress is widely recognized as a critical pathological mechanism in the development of liver injury and plays a central role in CCl4-induced hepatic damage [29]. CCl4 significantly elevates ROS levels, and excessive ROS can damage cellular lipids, proteins, and DNA, trigger apoptosis, and ultimately lead to liver dysfunction and injury [30]. MDA, a key end-product of lipid peroxidation, serves as an important biomarker of oxidative stress [31]. Under oxidative conditions, intracellular levels of ROS such as superoxide anions (O_2_^−^) and hydrogen peroxide increase, attacking lipid molecules and initiating peroxidation processes that culminate in MDA formation [32]. The liver contains an abundance of antioxidant enzymes, including SOD and glutathione (GSH), which play a vital role in neutralizing O_2_^−^, limiting lipid peroxidation, and maintaining cellular homeostasis as the body’s primary line of antioxidant defense [33]. In our study, CCl4 exposure markedly reduced SOD and GSH-Px activities while increasing MDA levels in mouse liver tissue, whereas GACE treatment significantly elevated hepatic SOD and GSH-Px activities, thereby exerting a protective effect against oxidative liver damage. In this experiment, CCl4 significantly reduced the antioxidant function of mice. After GACE intervention, the levels of SOD and GSH-Px in liver tissue were significantly increased, exerting a protective effect against oxidative damage in liver tissue, which may be related to the active components in GACE. Tangpradubkiat’s study showed that geniposide could significantly reduce MDA content in liver tissue and increase SOD and GSH levels, thereby improving APAP-induced liver injury through antioxidant pathways [11]. *Astragalus polysaccharides* in GACE play an important role in treating liver injury by regulating bile acids (BA) and increasing the serum level of thio-β-hydroxy-β-methyl-δ-cholic acid [34]. In addition, chlorogenic acid in GACE also has antioxidant effects. Chlorogenic acid can trigger the expression of PGC-1α by activating Nrf2, further enhancing PPARα-mediated fatty acid β-oxidation, thereby promoting liver regeneration after CCl4 poisoning [35].

ALT and AST are key intracellular enzymes found in hepatocytes, and when liver cells are damaged, these enzymes are released into the bloodstream; their serum concentrations positively correlate with the severity of liver injury, making them essential biomarkers for assessing liver function. In our CCl4-induced liver injury model, serum ALT and AST levels were significantly elevated in the model group compared to the control group, indicating substantial hepatocellular damage. Treatment with GACE effectively reduced serum ALT and AST levels in a dose-dependent manner, with the highest efficacy observed at a dosage of 6 g/kg. High-performance liquid chromatography (HPLC) analysis confirmed that GACE contains abundant geniposide and chlorogenic acid, both of which are known for their hepatoprotective properties [36]. Li et al. reported that geniposide confers liver protection in rats by modulating PPAR, MAPK, and apoptotic signaling pathways, suppressing inflammation, and reducing apoptosis [37], while Shi et al. found that chlorogenic acid mitigates CCl4-induced acute liver injury by inhibiting activation of the NLRP3 inflammasome and downregulating the expression of NLRP3, Pro-Caspase-1, Caspase-1, Pro-IL-1β, and IL-1β, along with lowering serum levels of TNF-α, IL-6, IL-1β, and hepatic mRNA expression [38]. Inflammatory cytokines, which regulate immune and inflammatory responses, are broadly categorized into pro-inflammatory and anti-inflammatory types; in response to external stimuli, the body secretes various cytokines, including interleukins, chemokines, interferons, and tumor necrosis factors, to modulate immune cell activity. Previous studies have demonstrated that hepatic inflammation and oxidative stress stimulate the release of key cytokines such as IL-1β, TNF-α, IL-6, and IL-10, all of which play pivotal roles in liver pathology [39]. In the context of CCl4-induced liver injury, CCl4 exacerbates hepatic damage by modulating the activity of inducible enzymes, which in turn promotes the release of downstream inflammatory cytokines [40]. In our study, GACE administration significantly suppressed the secretion of pro-inflammatory cytokines IL-2 and IL-12 while enhancing the production of the chemokine CCL8, indicating that its hepatoprotective effects may be mediated, at least in part, through the regulation of inflammatory cytokine expression.

The phosphatidylinositol 3-kinase (PI3K)/protein kinase B (AKT) signaling pathway is involved in numerous pathological and physiological processes related to liver injury and represents a critical target for regulating acute liver damage [41]. PI3K, a member of the phosphatidylinositol kinase family, generates phosphatidylinositol (3,4,5)-trisphosphate (PIP3), which subsequently activates AKT through phosphorylation, thereby influencing key cellular processes such as proliferation, growth, survival, and apoptosis [42,43,44]. This pathway plays essential roles in combating hepatic fibrosis [45], suppressing the proliferation and migration of HCC cells [46], and alleviating hepatic ischemia–reperfusion injury [47], acute liver injury [48], and acute-on-chronic liver failure [49]. In our study, transcriptomic analysis combined with WGCNA predicted that GACE exerts regulatory effects on the PI3K-AKT signaling pathway by enhancing the phosphorylation of PI3K and AKT in CCl4-induced liver injury. These predictions were supported by experimental validation, which confirmed that GACE significantly mitigates CCl4-induced hepatic injury through modulation of the PI3K signaling cascade.

The mitogen-activated protein kinase (MAPK) signaling pathway is a key intracellular cascade that regulates the expression of various cytokines and is closely linked to inflammatory responses associated with liver injury. This pathway comprises three main components: extracellular signal-regulated kinases (ERK), p38 kinases, and c-Jun N-terminal kinases (JNK). ERK1/2, a downstream effector of the epidermal growth factor receptor (EGFR)-mediated pathway, controls the production of multiple inflammatory cytokines and plays a critical role in the progression of HCC [50]. JNK, another major component of the MAPK pathway, phosphorylates substrates such as JUN and has been implicated in acetaminophen (APAP)-induced liver injury [51,52]. In our study, GACE administration significantly downregulated the expression of phosphorylated p38, JNK, and ERK proteins in CCl4-induced liver injury, indicating that GACE mitigates hepatic damage by modulating the MAPK signaling pathway.

This study demonstrates that GACE effectively alleviates hepatic pathological damage, oxidative stress, and inflammation, reduces hepatocyte apoptosis, and exerts a significant therapeutic effect on liver injury by modulating the PI3K-Akt and MAPK signaling pathways. However, as a preclinical basic study, it possesses certain limitations. First, there are significant differences between ICR mice and humans in terms of genetics, metabolism, and immunity, resulting in the inability to directly apply efficacy and safety data from mouse models to humans. Second, the CCl4-induced chemical acute liver injury model is suitable for drug screening and preliminary mechanistic studies; however, the etiology of clinical liver injury is complex and includes multiple types, such as NAFLD or cirrhosis. This study did not investigate the effects of GACE on liver injury from these different etiologies, and its generalizability requires further validation.

## 5. Conclusions

This study systematically revealed that the GACE exhibits significant protective effects against CCl4-induced liver injury in mice. Experimental results demonstrated that GACE could dose-dependently reduce serum ALT and AST levels, alleviate histopathological damage in liver tissue, and decrease hepatocyte apoptosis (with a significant reduction in TUNEL-positive cell rate). Simultaneously, GACE effectively mitigated CCl_4_-induced oxidative stress by enhancing SOD and GSH-Px activities in liver tissue while reducing MDA content. It also downregulated pro-inflammatory cytokines IL-6, IL-12, and IL-2, upregulated the anti-inflammatory cytokine IL-10, and suppressed inflammatory responses. Through transcriptome analysis combined with WGCNA and metabolomics, it was identified that the PI3K/AKT and MAPK signaling pathways are the key pathways through which GACE exerts its hepatoprotective effects. Western blot validation confirmed that this effect is achieved by inhibiting the phosphorylation of PI3K, AKT, p38, JNK, and ERK1/2 proteins. This study not only provides solid experimental evidence for GACE as a natural product combination in treating chemical-induced liver injury, but also reveals its molecular mechanism of synergistic anti-inflammatory and antioxidant effects through the regulation of multiple signaling pathways, enriching the modern pharmacological understanding of “medicinal and edible homology” in traditional Chinese medicine formulations. GACE has the potential to be developed as a novel hepatoprotective drug or functional food, particularly suitable for early intervention and adjuvant therapy in chronic liver injury. Future research will focus on isolating and identifying the key active monomers in GACE, elucidating their structure–activity relationships; validating their universal applicability in more clinically relevant models such as alcoholic or non-alcoholic fatty liver disease and drug-induced liver injury; and conducting long-term toxicology and pharmacokinetic studies to evaluate their safety, thereby laying the foundation for clinical translation.

## Data Availability

The original contributions presented in this study are included in the article. Further inquiries can be directed to the corresponding author.
